# Prussian Blue Anchored on Reduced Graphene Oxide Substrate Achieving High Voltage in Symmetric Supercapacitor

**DOI:** 10.3390/ma17153782

**Published:** 2024-08-01

**Authors:** Lindiomar Borges Avila, Pablo A. Serrano, Luis Torres Quispe, Adriana Dantas, Diogo Pontes Costa, Edy Elar Cuevas Arizaca, Diana Patricia Paredes Chávez, César Daniel Valdivia Portugal, Christian Klaus Müller

**Affiliations:** 1 Departamento de Física, Universidade Federal de Santa Catarina, Florianópolis 88040-900, Brazil; lindiomarbaj@gmail.com (L.B.A.); pablo.serrano.a@posgrad.ufsc.br (P.A.S.); diogopontes102@gmail.com (D.P.C.); 2 Escuela Profesional de Física, Universidad Nacional de San Agustín de Arequipa, Av. Independencia s/n, Arequipa 04000, Peru; l.torre.q@gmail.com; 3 Food Processing and Engineering Programme, Institute of Agrifood Research and Technology (IRTA), 17121 Monells, Spain; adriana.dantas@irta.cat; 4 Vicerrectorado de Investigación, Universidad Católica de Santa María, Arequipa 04000, Peru; ecuevas@ucsm.edu.pe (E.E.C.A.); dparedesch@ucsm.edu.pe (D.P.P.C.); cvaldiviap@ucsm.edu.pe (C.D.V.P.); 5 Faculty of Physical Engineering/Computer Sciences, University of Applied Sciences Zwickau, 08056 Zwickau, Germany

**Keywords:** Prussian blue, graphene, supercapacitor electrode, symmetric supercapacitor

## Abstract

In this work, iron hexacyanoferrate (FeHCF—Prussian blue) particles have been grown onto a reduced graphene oxide substrate through a pulsed electrodeposition process. Thus, the prepared FeHCF electrode exhibits a specific volumetric capacitance of 88 F cm^−3^ (specific areal capacitance of 26.6 mF cm^−2^) and high cycling stability with a capacitance retention of 93.7% over 10,000 galvanostatic charge–discharge cycles in a 1 M KCl electrolyte. Furthermore, two identical FeHCF electrodes were paired up in order to construct a symmetrical supercapacitor, which delivers a wide potential window of 2 V in a 1 M KCl electrolyte and demonstrates a large energy density of 27.5 mWh cm^−3^ at a high power density of 330 W cm^−3^.

## 1. Introduction

As a result of the depletion of fossil fuels and the subsequent increase in air pollution, there is an urgent demand for clean and alternative energy sources. Consequently, significant efforts have been directed towards harnessing sustainable energy sources, such as solar and wind energy systems. Nonetheless, the intermittent nature of these renewable energy sources hinders their substantial impact unless the electricity they generate is efficiently stored. Hence, there arises a critical need to employ advanced energy storage devices to effectively store the energy produced by renewable sources. Supercapacitors, also referred to as electrochemical capacitors, have garnered significant interest as innovative energy storage systems, thanks to their exceptional attributes, such as high-power densities, extended lifespan, and rapid charging capabilities, setting them apart from conventional batteries [1,2]. The supercapacitor material is the core component of supercapacitor electrodes, which largely dictates their electrochemical performance. A variety of electrode materials have been investigated for supercapacitor electrodes, including carbon materials [3], polymeric materials [4], and metal oxides [5]. In 2008, Chen et al. [6] achieved the successful synthesis of three distinct types of transition metal hexacyanoferrates (FeHCF, NiHCF, and CoHCF). These compounds were employed as active electrodes in a supercapacitor operating with a 1 M KNO_3_ electrolyte. Among the tested materials, FeHCF (425 Fg^−1^), NiHCF (574.7 Fg^−1^), and CoHCF (261.56 Fg^−1^) electrodes demonstrated higher discharge capacities at a current density of 0.2 Ag^−1^. Composites of transition metal hexacyanoferrates with other components have also been used for supercapacitor electrodes. Prussian blue analogs are promising candidates for storing various ions in aqueous electrolytes, where longevity and cycling stability are given utmost importance to maintain economic viability. Long-term cycling of PBAs has recently been successfully demonstrated by the use of highly concentrated aqueous electrolytes [7,8].

There are several concerns that need to be further improved in supercapacitors, for example, to extend the working voltage, promote the energy/power densities, long lifespan, lower the construction cost, and their environmental benign nature [6]. Asymmetric cell configuration is considered an effective way to extend the voltage window of the cell, where positive electrodes (metal oxide-based) and negative electrodes (carbon-based) are generally used [9]. Recent studies have indicated that metal oxides (pseudocapacitive materials) could also be used as the negative electrode in asymmetric supercapacitors [10,11]. For example, Hu et al. [12] have reported Fe_2_O_3_ nanoparticle cluster/reduced graphene oxide (rGO) paper as a negative electrode that shows improved capacitance in the negative voltage range compared to pristine rGO paper. In another work, Zhang et al. [13] have reported MoO_2_/MoS_2_ as a negative electrode that shows good electrochemical performance. Ahmed Galel and his group [14] presented an extensive electrochemical study of the SRGO supercapacitor in redox systems containing electrolytes. They explored the influence of a redox electrolyte on the specific capacitance of SrRuO_3_/rGO nanocomposites. Another work displays the exceptional electrochemical performance of the polypyrrole/reduced graphene oxide (PPy/rGO-HT) composite electrode within an aqueous electrolyte enriched with hydroquinone redox-active species [15]. The electrode design has significantly pushed the boundaries of what symmetric supercapacitors can achieve. Notably, Xia et al. and other groups have developed a high-voltage symmetric supercapacitor capable of reaching a cell voltage of up to 1.6 V [16,17,18]. This achievement marks a substantial improvement over traditional symmetric supercapacitors and opens new possibilities for their application in energy storage devices. Moreover, a breakthrough study has shown that incorporating rGO can further enhance the performance of these devices [19]. Latter work has managed to extend the potential window up to 1.8 V, setting a new benchmark for aqueous symmetric capacitors. M. B. Arvas successfully synthesized a polypyrrol (PPy)/hydroxy naphtol blue (HNB) electrode, achieving a working potential window of 2.5 V in an aqueous symmetric supercapacitor using a two-electrode configuration [20]. Although this result is remarkable, the durability is limited to only 5000 cycles.

Nevertheless, designing pseudocapacitive materials as the negative electrode is not simple and often leads to poor electrochemical performance and instability [21]. In 2014, a supercapacitor based on activated carbon showcased remarkable stability during operation, maintaining an unexpectedly high cell voltage of 2.2 V [22]. By manipulating the physicochemical properties of the electrolyte (such as the pH or conductivity values) and/or adjusting the surface chemistry of activated carbons, it becomes feasible to elevate the maximum voltage of aqueous supercapacitors. These modifications directly influence the over-potential for water splitting [23]. Aqueous electrolyte-based supercapacitors have garnered attention owing to their favorable attributes, such as relatively high capacitance, excellent power rates, affordability, and eco-friendliness. However, as they are commonly operated in highly corrosive electrolytes, there is a risk of corrosion damage to their closures. To enhance safety, it is preferable to design electrodes suitable for neutral electrolytes [24]. Thus far, only carbonaceous materials and manganese oxide (MnO_2_)-based electrode materials have shown good electrochemical performance in neutral media [25,26]. However, some issues persist with these materials, e.g., carbon-based electrodes suffer from relatively low capacitance, and MnO_2_-based electrodes are restricted in their cycling stability [27]. Recently, Zan et al. reported about mesoporous cubic cages assembled by Ni(OH)_2_-coupled monolayers intercalated with VO_4_^3–^ (NiCMCs) with a high capacity and long cycling [28].

Prussian blue (PB) and its analogs, due to their diverse morphologies and easily controllable size, have received considerable attention in electrochemical devices, such as electrochemical biosensors [29,30], memristor devices [31,32], and also for supercapacitors [33,34,35,36]. The general formula of PB and its analogs is given by $A_{x}M_{y}^{a}{\left[M^{b}{\left(CN\right)}_{6}\right]}_{z}$, where the A positions are occupied with alkali metal ions, and the M positions correspond to transition metal ions. The open framework structure of FeHCF (A = K^+^, M = Fe^2+^, Fe^3+^) has wide channels that allow the rapid insertion and removal of electrolyte ions through a redox reaction between ferrous and ferric oxidation states in the Fe center [37,38,39]. The rich intercalation chemistry of FeHCF, its high charge conductivity, and high surface area have motivated us to explore this unique material for supercapacitor applications. Among other preparation methods, electrodeposition is highly suitable to obtain binder-free electrodes of PB with enhanced capacitance [40]. In practice, symmetrical supercapacitors are limited to the voltage range of ~1.23 V in aqueous electrolytes. In this work, we have grown FeHCF particles onto a reduced graphene oxide substrate using pulsed electrodeposition for supercapacitor electrodes. These techniques allow us to produce uniform FeHCF layers with defined physical properties. Furthermore, a symmetrical supercapacitor was constructed by pairing up two identical electrodes and achieving a high operating voltage of 2 V with a high energy density and power density. This electrode material shows large volumetric and areal capacitance and ultrahigh cyclic stability in a 1 M KCl electrolyte. Additionally, the chemical stability and low cost of FeHCF make this material very attractive for practical application in supercapacitors.

## 2. Materials and Methods

### 2.1. Sample Preparation

**Figure 1:**
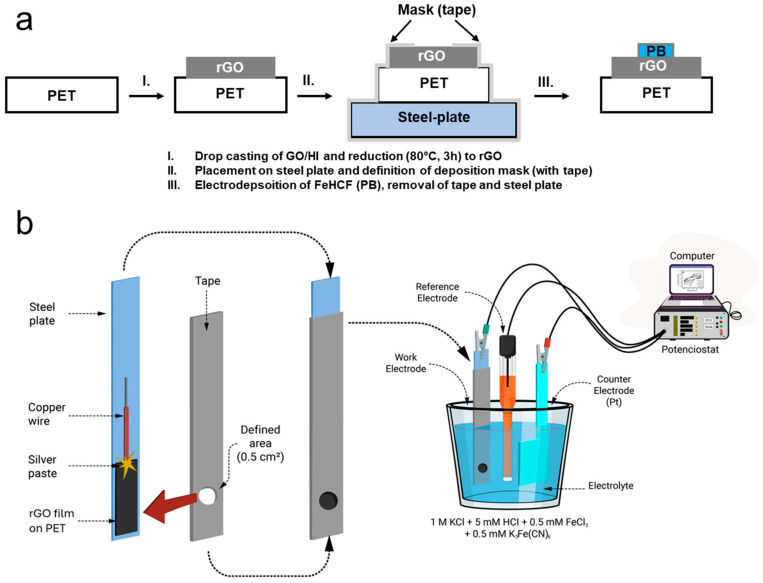
Schematic illustration of the fabrication process of rGO/FeHCF films. (**a**) Fabrication scheme with principal fabrication steps, (**b**) setup for electrodeposition of FeHCF on rGO.

Firstly, we placed a rGO film as a conductive substrate onto a polyethylene terephthalate (PET) sheet in the following way: the suspension of GO (2 mg mL^−1^, Sigma Aldrich, Darmstadt, Germany) was mixed with HI (57%) with a volume ratio of 2:0.5. Then, the mixture was dropped onto the pre-cleaned PET sheet. After that, the PET sheet was heated up directly by a hot plate at the temperature of 80 °C for 3 h. The obtained rGO was washed with a copious amount of double distilled water and then ethanol to remove residual iodine. The prepared substrates were preserved for the further electrodeposition of FeHCF (see Figure 1a). The rGO substrates were electrically contacted by a copper wire and masked to define the area for the electrodeposition of FeHCF. Then, it was immersed in the aqueous electrolyte composed of 0.5 mM of FeCl_3_ (ACS, 98–102%, Sigma Aldrich, Darmstadt, Germany), 0.5 mM of K_3_Fe(CN)_6_ (ACS, >99%, Sigma Aldrich, Darmstadt, Germany), 1 M of KCl (ACS, 99–100.5%, Sigma Aldrich, Darmstadt, Germany), and 0.01 M of HCl (ACS, 37%, Sigma Aldrich, Darmstadt, Germany). With the chosen electrolyte composition, a sufficiently high enough conductivity for fast film deposition could be achieved. The electrochemical deposition of the FeHCF films was performed in potentiostatic mode using an electrochemical workstation (Ivium CompactStat, Eindhoven, Netherlands), as shown in Figure 1b. The rGO was used as the working electrode, while the counter and reference electrodes were a platinum foil and a saturated calomel electrode (SCE), respectively. To electroplate Fe-HFC onto the rGO substrate, pulses of potential were applied, first a pulse at 0.3 V, followed by pulses at 0 V, −0.3 V, and 0 V vs. SCE. All pulses were kept with the same width of 0.1 s. Such a process was performed repeatedly 300 times. After this, the samples were washed with water and dried under a stream of nitrogen gas. The aqueous symmetric supercapacitor (two-electrode cell system) was made up by pairing two identical electrodes of FeHCF separated by filter paper in an aqueous 1 M KCl electrolyte. The symmetrical supercapacitor system was operated by cyclic voltammetry over a wide voltage range of 0–2 V at a constant scan rate of 50 mV/s. The electrochemical process uses only small amounts of chemicals, and several depositions could be performed with the same electrolyte. It is important to mention that to increase the device capacity, the electrochemical sample fabrication could be applied to larger substrate areas.

### 2.2. Sample Analysis

The film morphology was analyzed by field emission scanning electron microscopy (FEG-SEM, TESCAN CLARA, Brünn, Czech Republic) at 5 kV. The elemental composition was studied with an energy-dispersive X-ray (EDX) detector (Ultim Max 65 SDD, Oxford Instruments, Wiesbaden, Germany) at 15 kV. The crystallographic film structure was analyzed with a Siemens X-ray diffractometer (D5000, Siemens, München, Germany) using Bragg–Brentano geometry, a Cu Kα X-ray source (λ = 1.5418 Å), 1 mm slit size (for incident and diffracted beam), and a scintillator detector. With the three electrodes (see Figure 1b), the specific capacitance values can be determined from galvanostatic charge–discharge curves (GCDs). From the GCDs, the specific areal capacitance (C_S,A_, mF cm^−2^) and the specific volumetric capacitance (C_S,V_, mF cm^−3^) were calculated according to the following Equations (1) and (2):

(1) $$C_{S,A}=\frac{I\cdot \Delta t}{A\cdot \Delta V}$$

(2) $$C_{S,V}=\frac{I\cdot \Delta t}{V\cdot \Delta V}$$

where I is the applied constant current, Δt is the discharge time, A is the active area, V is the active volume, and ΔV corresponds to the discharge time. Furthermore, the values of energy density (E_S_, mWh cm^−3^) and power density (P_S_, W cm^−3^) can be obtained from Equations (3) and (4):

(3) $$E_{S}=\frac{1}{2}C_{S}\cdot {\Delta V}^{2}$$

(4) $$P_{S}=\frac{E_{S}}{\Delta t}.$$

## 3. Results and Discussion

**Figure 2:**
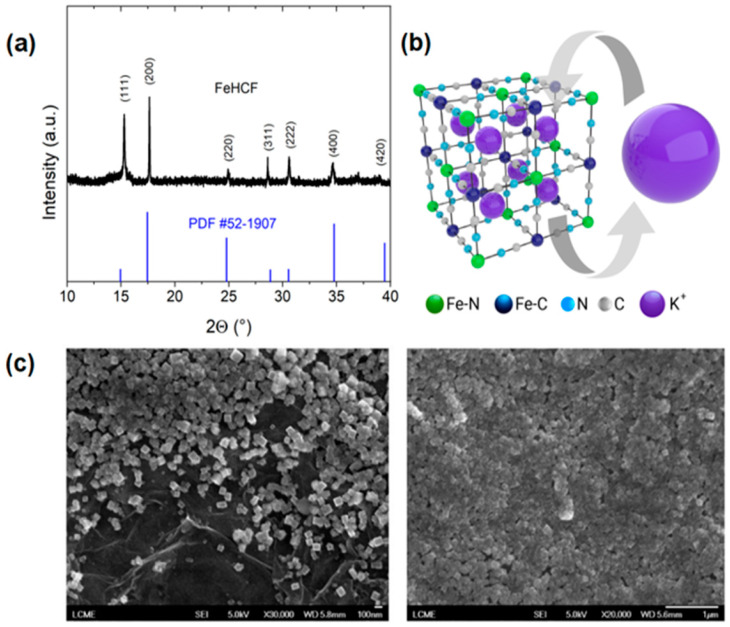
Structure and morphology of FeHCF. (**a**) Comparison of XRD pattern of FeHCF nanoparticles with PDF of FeHCF, (**b**) demonstration of a unit cell of cubic FeHCF and insertion/exertion of K^+^ ion, and (**c**) SEM images of as-prepared FeHCF nanoparticles onto rGO.

The structure analysis of the as-prepared FeHCF was performed by X-ray diffraction, as presented in Figure 2a. Almost all diffraction peaks were indexed to the face-centered Prussian blue structure according to the JCPDS Card No. 52-1907, indicating a crystalline cubic structure of FeHCF particles, with a lattice constant of a = 10.15 Å (for the 200, 220, and 400 reflexes) in agreement with the literature [41]. The peak at 25° was assigned due to graphene plane stacking diffraction [42]. PB with the formula KFe(III)[Fe(II)CN_6_] is the partially reduced counterpart whereby the Fe center coordinated by C has its oxidation state +2, due to the strong field of the CN ligand. On the other hand, in the peripheral cyanide of coordination N, Fe-N, the Fe atoms are in the +III oxidation state and with their tetrahedral holes occupied by alkaline cations (see Figure 2b). Figure 2c presents an SEM image of the electrodeposited FeHCF onto the graphene substrate, one region showing the FeHCF anchored in the rGO (Figure 2c, left image) and the other with the most agglomerated particles covering the entire electrode (Figure 2c, right image).

**Figure 3:**
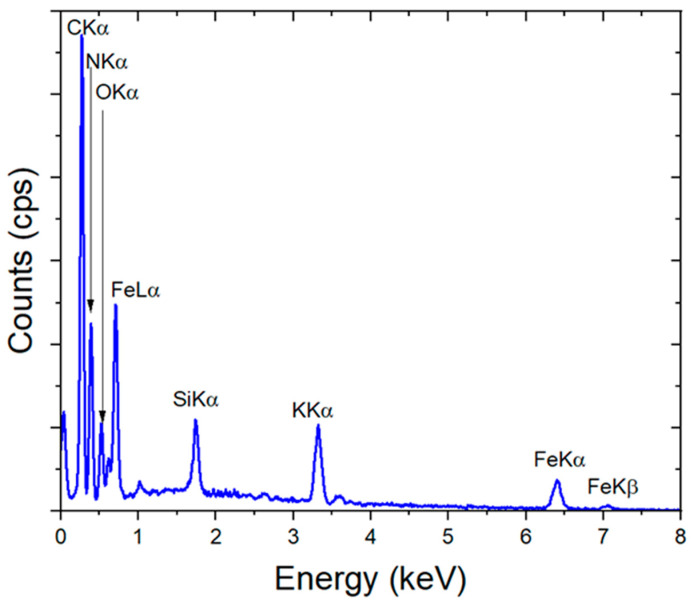
EDX analysis of FeHCF on rGO measured at 15 keV.

EDX analysis was performed to determine the layer composition (see Figure 3). From the EDX spectrum, a composition with 42 at% C, 35 at% N, 6 at% O, 12.5 at% Fe, and 4.5 at% Na is obtained. From these amounts, a mixture of 92% KFe^3+^[Fe^2+^(CN)_6_]·mH_2_O and 8% Fe^3+^_4_[Fe^2+^(CN)_6_]_3_·mH_2_O of PB can be proposed based on the found C/N ratio of ~1.2 and an Fe/Na ratio of ~2.6. The higher amount of C compared with N occurs because of carbon from the rGO and surface contaminations.

**Figure 4:**
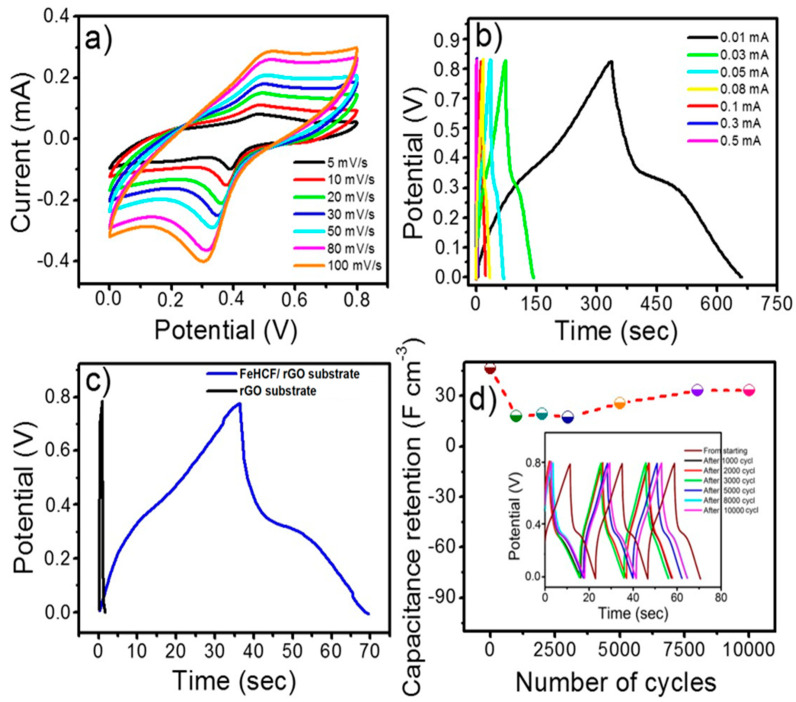
Electrochemical characterization of FeHCF electrode in positive potential range in three-electrode cell system. (**a**) Cyclic voltammogram curves of FeHFC onto rGO substrate in the positive regions for different scan rates, (**b**) galvanostatic charge–discharge curves at different current densities, (**c**) difference in charge–discharge curve of bare rGO substrate and FeHCF/rGO substrate at a current density of 0.05 mA cm^−2^, and (**d**) cycling behavior of FeHCF electrode at different cycle numbers at a current density of 0.1 mA cm^−2^ (inset shows the charge–discharge curve right after being taken at different hundreds of cycles).

The electrochemical performance of FeHCF as a supercapacitor electrode was analyzed through comprehensive cyclic voltammetry (CV) and precise galvanostatic charge–discharge (GCD) measurements, conducted within a sophisticated three-electrode cell system. In this carefully designed setup, a highly conductive 1 M KCl electrolyte was employed, while a Pt foil and saturated calomel electrode were meticulously chosen as the counter and reference electrodes, respectively, ensuring accurate and reliable characterization of the FeHCF electrode’s behavior. In Figure 4a, a distinct pair of reversible peaks is evident, corresponding to the redox reactions of FeII/III within FeHCF. These reactions are accompanied by the insertion of K+ ions to maintain charge neutrality [43]. Furthermore, as the scan rates increase, there is a notable rise in the electrochemical response current. The cyclic voltammetry (CV) curves exhibit a nearly consistent reversible pattern with slight shifts in peak positions, indicating excellent electronic conduction within the electrode. The faradaic transition of PB in the presence of K+ ions is proposed as follows [44].

Fe^(III)^_4_[Fe^(II)^(CN)_6_]_3_ + 4K^+^ + 4e^−^ = K_4_Fe^(II)^_4_[Fe^(II)^(CN)_6_]_3_ (5)

For further investigation of the capacitive behavior of the FeHCF electrode, a series of GCD measurements were performed at various current densities, as shown in Figure 4b. The discharge curves of the FeHCF electrode displayed a distinct plateau, providing clear evidence of its pseudocapacitive behavior, which aligns closely with the observations from the cyclic voltammetry analysis. Areal and volumetric capacitances of the electrode were calculated from the discharge curves at different current densities and the results are listed in Table 1. At the highest volumetric current density of 3.3 A cm^−3,^ the charge or discharge step was completed in less than 6 s. To eliminate any potential influence from the graphene substrate as a working electrode, we conducted charge–discharge measurements on both the pure rGO substrate and the FeHCF/rGO substrate, under identical conditions. The experiments were carried out at a current density of 0.33 A cm^−3^. The results revealed a significantly lower capacitance for the pure graphene substrate, as illustrated in Figure 4c. The cyclic stability of the electrode materials is another important parameter in the selection of supercapacitor electrodes. The cycling stability of the FeHCF was performed under repetitive GCD cycles at the constant volumetric current density of 0.33 A cm^−3^. The variation in capacitance with cycle number is shown in Figure 4d. Initially, a gradual decline in capacitance was observed, followed by a notable surge after 2500 cycles, indicative for the activation process of the electrode material. This phenomenon suggests that over time, the electrode material undergoes a maturation process, leading to enhanced performance. The prolonged duration of the charging–discharging cycle likely facilitates the intercalation of electrolyte ions into the porous structure of FeHCF nanoparticles, thereby maximizing the utilization of the available surface area of the electrode material. The phenomenon observed in the structure of FeHCF is widely recognized, as its open framework features expansive channels facilitating the swift insertion and extraction of electrolyte ions. This process occurs through redox reactions involving the conversion between ferrous and ferric oxidation states at the Fe center. Extensively documented in the literature, this characteristic has been thoroughly investigated and discussed in various studies [37,38,39]. After repeating 10,000 GCD cycles, more than 93% capacitance was retained from its initial one, indicating the ultrahigh stability of the electrode materials in a 1 M KCl electrolyte (pH~5). Most of the works performed with Prussian blue analogs reported a lower number of cycles [1]. This long-term cyclic stability of FeHCF is probably due to the strong interaction of FeHCF nanoparticles at the interface of the graphene substrate. Cycling stability behavior is the foremost metric determining the success of supercapacitor electrode materials. The inset of Figure 4d shows that the GCD curves taken after 1000, 2000, 3000, 5000, 8000, and 10,000 cycles have no significant change in the symmetry of the charge and discharge curves after consecutively repeating cycles. Again, the electrode material is demonstrating a long-term reliability of the electrochemical performance. The stability of the electrode materials is also dependent on the choice of electrolyte. Generally, Prussian blue analogs are known to be more stable in acidic electrolytes than in neutral and basic media. The above findings reveal that FeHCF nanoparticles have a large specific capacitance and excellent cycling reliability in a KCl electrolyte at ~pH 5.

**Figure 5:**
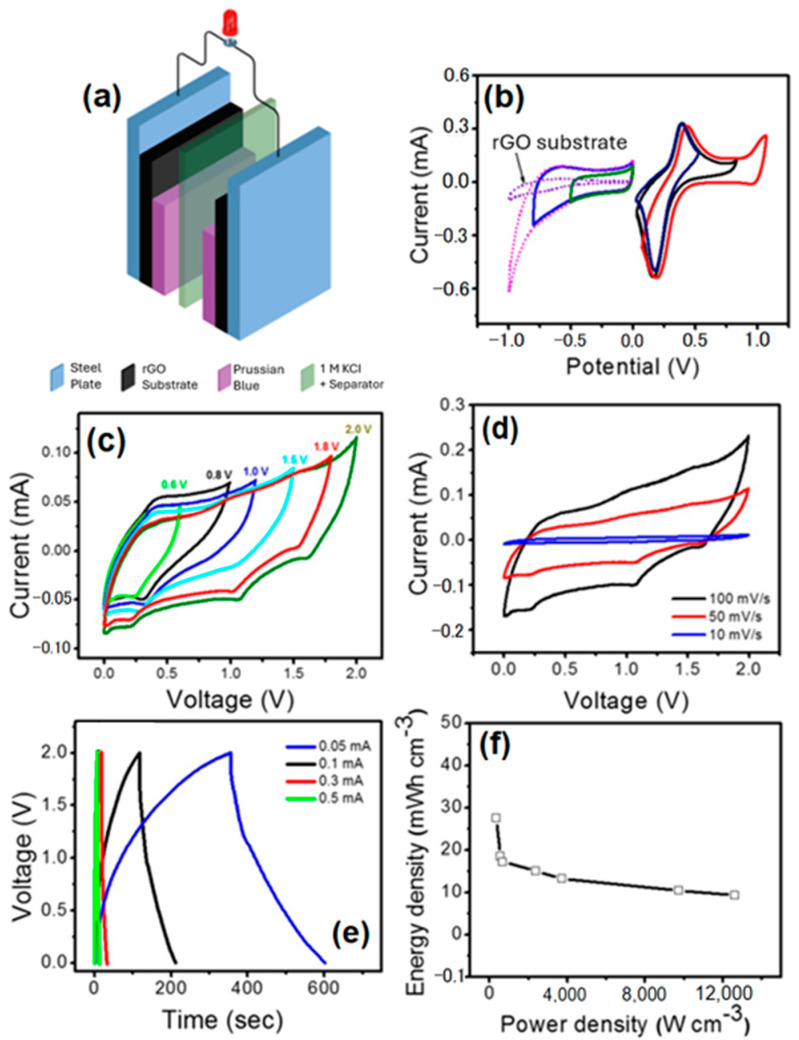
Symmetrical supercapacitor performance. (**a**) Schematic illustration of the fabricated symmetrical supercapacitor device, (**b**) cyclic voltammogram behavior of graphene substrate alone as well as FeHCF onto rGO substrate in negative and positive potential windows, (**c**) cyclic voltammogram curves of symmetrical supercapacitor at different voltage windows, (**d**) CV curves of symmetrical supercapacitor at different scan rate in a 2 V voltage window, (**e**) galvanostatic charge–discharge curves at different current densities, and (**f**) Ragone plot, energy density versus power density.

The symmetrical supercapacitor was mounted as shown in Figure 5a. Before the pairing of two identical electrodes with the same shape and size, we first tested the FeHCF electrode in negative and positive potential windows under different potential ranges in a three-electrode cell setup and at a scan rate of 100 mV s^−1^. As shown in Figure 5b, the CV curves of the FeHCF electrode exhibit a rectangular shape without any redox peaks in the negative potential region, which indicates pure capacitive behavior. The rectangular shape of the pseudocapacitive materials in the negative windows has also been shown in earlier reported works [45]. The pseudocapacitive behaviors of PB and its analogs involve a hybrid ion storage and intercalation mechanism. These mechanisms have been extensively studied, but the depth of the surface charge layer remains unknown [46]. However, to rule out the capacitive behavior of the FeHCF electrode in the negative potential window, we measured the CV of the rGO substrate without FeHCF from 0 to −1 V. The CV curve of the pure rGO substrate was smaller than with FeHCF; this means that rGO substrate has a lower capacitance than the FeHCF-coated rGO substrate in the negative potential window (Figure 5b). Thus, it is clear that in FeHCF, the additional capacitance is arising from FeHCF showing typical electrical double-layer capacitance behavior in negative potential windows with the KCl electrolyte. Furthermore, the current leap of the FeHCF electrode at the negative end (~1 V) of the potential window increases sharply, which indicates hydrogen evaluation in the KCl electrolyte. A sharp increase in current for the FeHCF electrode near ~1 V is due to the oxygen evaluation on the electrode.

According to the performance of FeHCF in negative and positive potential windows in a three-electrode cell configuration, a symmetrical supercapacitor (two-electrode cell system) was made up by pairing two identical electrodes of FeHCF separated by filter paper in an aqueous 1 M KCl electrolyte. The symmetrical supercapacitor system was operated by cyclic voltammetry over a wide voltage range of 0–2 V at a constant scan rate of 50 mV/s, as shown in Figure 5c. Figure 5d shows the CVs of the full cell between 0 and 2 V at various scan rates. The specific volumetric capacities of a symmetrical supercapacitor of 49.5 F cm^−3^ at a volumetric current density of 0.06 A cm^−3^ and of 6.4 F cm^−3^ at a volumetric current density of 3.3 A cm^−3^ were recorded from the discharge curve, as shown in Figure 5e. He et al. reported values of up to 4.77 F cm^−2^ and 347 F cm^−3^ for pyridine-modified PB thin films with voltages up to 1 V [47]. It is worth mentioning that our symmetrical supercapacitor successfully achieved 2 V of the voltage window in two-electrode cell configurations. Most symmetrical supercapacitors are typically limited to a voltage range of approximately 1 V [16,17,48]. However, Xia et al. developed a high-voltage symmetric RuO_2_//RuO_2_ supercapacitor that achieves a cell voltage of up to 1.6 V [16]. More recently, another study utilizing rGO succeeded in achieving a potential window of up to 1.8 V [19]. These advancements illustrate that depositing PB on the rGO surface is crucial for expanding the working potential window.

The device prepared here demonstrates the energy density of 27.5 mWh cm^−3^ at the power density of 330 W cm^−3,^ which is larger than in [47], where up to 12.1 mWh cm^−3^ were achieved. The energy density can be maintained at 9.3 mWh cm^−3^ with a power density of 12,600 W cm^−3^ (Figure 5f). This characteristic is notable in that a high power density can be simultaneously achieved along with a high specific energy, thus making these materials very promising for high-energy storage supercapacitors. The good electrochemical performance of the FeHCF electrode material in the aqueous electrolyte may be ascribed to the following factors: firstly, the open framework of FeHCF contains large interstitial sites that can host large amounts of potassium ions that can be transported within the channels. Secondly, the high ionic conductivity of the aqueous electrolyte may facilitate the fast ionic transport between the electrolyte and the electrode.

## 4. Conclusions

In summary, iron hexacyanoferrate nanoparticles have been grown onto a rGO substrate using pulsed electrodeposition and used as a supercapacitor electrode. The electrode demonstrates exceptional electrochemical performance, excelling in both capacitance and stability aspects. This electrode delivers a high specific volumetric capacitance of 88 F cm^−3^ (specific areal capacitance of 26.6 mF cm^−2^) and maintains the capacitance at about 93.7% after repeating 10,000 galvanostatic charge–discharge cycles in a 1 M KCl electrolyte. When two identical electrodes of FeHCF were paired up in the form of a symmetrical supercapacitor, it achieved a high operating voltage window of 2 V. Moreover, the symmetrical supercapacitor shows an energy density of 27.5 mWh cm^−3^ at a power density of 330 W cm^−3^.

## Figures and Tables

**Table 1:** Areal and volumetric capacitance of FeHCF at different current densities.

Current Density, *I/A* (mA cm^−2^)	Specific Areal Capacitance, $C_{S,A}$ (mF cm^−2^)	Volumetric Current Density, *I/V* (A cm^−3^)	Specific Volumetric Capacitance, $C_{S,V}$ (F cm^−3^)
0.01	26.6	0.06	88.0
0.03	17.1	0.19	63.0
0.05	14.0	0.33	46.4
0.08	12.8	0.52	37.2
0.1	8.5	0.66	23.7
0.3	6.3	1.98	15.8
0.5	3.9	3.30	12.3

## Data Availability

The original contributions presented in the study are included in the article, further inquiries can be directed to the corresponding author.
